# Interconnection between polycystic ovary syndrome, immune disorders, and reproductive outcomes

**DOI:** 10.1530/RAF-25-0186

**Published:** 2026-04-21

**Authors:** Thaís Bastos Romero, Daniel de Sousa Sobral, Lara Andryne Alves Aguiar, Helena Aben-Athar Ponte, Ricardo Espíndola Romero Filho, Ana Luiza Rangel Montenegro, Marla Girão Ramos Maia, Thiago Gonçalves Marques, Laís de Oliveira Martins, Marcelo Borges Cavalcante

**Affiliations:** ^1^ Graduate Program in Medical Sciences, Universidade de Fortaleza (UNIFOR), Fortaleza, CE, Brazil; ^2^ Medical School, Universidade de Fortaleza (UNIFOR), Fortaleza, CE, Brazil; ^3^ Veterinary Medicine School, Universidade de Fortaleza (UNIFOR), Fortaleza, CE, Brazil

**Keywords:** polycystic ovary syndrome, immune system diseases, immunology, pregnancy outcome

## Abstract

**Abstract:**

Polycystic ovary syndrome (PCOS) is a multifactorial disorder traditionally recognized for its endocrine and metabolic disturbances. However, accumulating evidence indicates that chronic low-grade inflammation and immune dysregulation play a central role in its pathophysiology. This narrative review explores the immunological mechanisms underlying PCOS and their repercussions for reproductive outcomes. Women with PCOS exhibit increased circulating levels of inflammatory cytokines – including interleukin (IL)-6, IL-1β, IL-18, and tumor necrosis factor-alpha (TNF-α) – alongside macrophage and lymphocyte infiltration within ovarian and endometrial tissues. These alterations promote oxidative stress, granulosa cell apoptosis, impaired steroidogenesis, and diminished endometrial receptivity. The adaptive immune response is similarly altered, characterized by imbalances between Th1/Th2 and Th17/regulatory T lymphocyte subsets and the presence of autoantibodies, including antinuclear, antithyroid, and antiphospholipid antibodies. These immune abnormalities have been associated with implantation failure and recurrent pregnancy loss. Furthermore, autoimmune diseases, including Hashimoto’s thyroiditis and type 1 diabetes mellitus, occur more frequently among women with PCOS, reinforcing the concept of an underlying systemic immunometabolic imbalance. Unlike prior reviews, this work offers a novel integrative framework that connects systemic inflammation with localized immune dysregulation in reproductive tissues, providing a more comprehensive understanding of PCOS pathophysiology from an immunological standpoint. This review also discusses the immunological implications of metabolic therapies – including metformin and glucagon-like peptide-1 receptor agonists – and the prospective role of targeted immunomodulation in selected immune-phenotypic PCOS subgroups. Understanding PCOS through an immunological lens provides a new framework for integrating metabolic, hormonal, and immune pathways, guiding future personalized interventions to improve reproductive outcomes.

**Lay summary:**

Polycystic ovary syndrome (PCOS) is one of the most common causes of infertility in women and involves not only hormonal and metabolic problems but also immune system dysregulation. Women with PCOS often experience chronic low-grade inflammation involving the ovaries and uterus, which can impair egg quality and hinder successful embryo implantation. They are also more prone to autoimmune diseases, including thyroid disorders, which can contribute to pregnancy complications and miscarriages. This review synthesizes recent studies showing how immune cells and inflammatory molecules interfere with fertility in PCOS and how certain existing treatments – such as metformin and glucagon-like peptide-1 receptor agonists – may also help by improving immune balance. Understanding these immune mechanisms will help researchers and clinicians develop more targeted and effective therapies, supporting women with PCOS in achieving healthier pregnancies.

## Introduction

Polycystic ovary syndrome (PCOS) is a heterogeneous endocrine disorder first clinically described in the 1930s. Its first formal characterization was conducted by Irving F Stein and Michael L Leventhal in 1935, when they reported seven women presenting with amenorrhea, hirsutism, and bilateral ovarian enlargement (Stein & Leventhal 1935). These clinical features were associated with multiple ovarian cysts identified through laparotomy. Their pioneering study, published under the title ‘Amenorrhea Associated with Bilateral Polycystic Ovaries’, marked the beginning of the clinical recognition of PCOS, which was initially referred to as ‘Stein–Leventhal syndrome’ (Stein & Leventhal 1935). Since then, the understanding of PCOS has evolved substantially, incorporating broader diagnostic criteria that encompass not only ovarian morphology but also hormonal and metabolic abnormalities.

PCOS is one of the most prevalent endocrinological disorders among women of reproductive age, affecting approximately 10–13% of the global female population (Bozdag *et al.* 2016, Joham *et al.* 2022). This multifactorial condition has a pathophysiology that remains incompletely understood. Insulin resistance (IR) and hyperandrogenism are key features in the pathogenesis of PCOS, which initiate various mechanisms that influence complex networks modulated by genetic, environmental, and epigenetic factors, including diet, physical activity, chronic stress, and exposure to endocrine-disrupting chemicals (Palioura & Diamanti-Kandarakis 2015, Escobar-Morreale 2018, Silva & Campbell 2022). Metabolic syndrome (MetS), obesity, type 2 diabetes, and nonalcoholic fatty liver disease are common comorbidities associated with PCOS, as is an increased risk of endometrial cancer (Apridonidze *et al.* 2005, Brower *et al.* 2013, Palomba *et al.* 2021, Ganie *et al.* 2024). The main clinical manifestations of PCOS include menstrual irregularities, hirsutism, acne, alopecia, weight gain, and infertility. The wide range of clinical presentations, primarily involving combinations of chronic anovulation, hyperandrogenism, and ovarian morphological alterations, contributes to the emergence of distinct phenotypes (Azziz *et al.* 2016, Baracat *et al.* 2022, van der Ham *et al.* 2024). Hormonal and metabolic disturbances, along with the considerable impact on physical appearance, increase the risk of behavioral and psychiatric disorders in women with PCOS, including anxiety and depression (Teede *et al.* 2010, Balen *et al.* 2024, Infante-Cano *et al.* 2025).

Several proposals have been made to standardize the diagnosis of PCOS. In 1990, the National Institutes of Health established the first diagnostic criteria for PCOS based on the presence of menstrual disturbances – such as oligomenorrhea or amenorrhea – associated with clinical and/or biochemical hyperandrogenism, independent of polycystic ovarian morphology on ultrasound examination (Teede *et al.* 2023). In 2003, with the Rotterdam Consensus, ovarian ultrasonography was incorporated as a diagnostic criterion, requiring two out of the following three criteria to confirm the syndrome: clinical or biochemical hyperandrogenism, ovulatory dysfunction, and polycystic ovarian morphology on ultrasound examination (Teede *et al.* 2023).

Given the central role of hyperandrogenism in PCOS pathophysiology, in 2006, the Androgen Excess and Polycystic Ovary Syndrome Society proposed the mandatory inclusion of hyperandrogenism as a criterion for PCOS diagnosis (Azziz *et al.* 2009). In 2018, an international consensus standardized the ultrasonographic criteria, defining it as the presence of at least one ovary containing ≥20 antral follicles. Phenotypic classifications were introduced based on different combinations of the diagnostic criteria met (Teede *et al.* 2023). The most recent international PCOS guideline (2023) introduced anti-Müllerian hormone (AMH) as an alternative marker for ovarian morphology and redefined ovarian volume (≥10 cm^3^) as an additional diagnostic variable. Importantly, PCOS remains a diagnosis of exclusion; conditions such as hypothyroidism, hyperprolactinemia, adrenal hyperplasia, and androgen-secreting tumors must be ruled out (Teede *et al.* 2023).

Inflammatory and immunological aspects have become increasingly evident in the pathophysiology of PCOS (Wang *et al.* 2023). In addition to an elevated risk of autoimmune diseases, women with PCOS exhibit chronic low-grade inflammation involving multiple systems, including the reproductive, endocrine, cardiovascular, digestive, and central nervous systems (Deng *et al.* 2024). The immune dysregulation observed in PCOS involves both innate and adaptive immunity, with a direct impact on reproductive outcomes. Regarding the immune cells, women with PCOS have elevated levels of macrophages, lymphocytes, eosinophils, neutrophils, monocytes, and T helper (Th) 17 cells and a reduced number of regulatory T (Treg) cells. The cytokines most associated with this condition include interleukin (IL)-6, IL-1, IL-18, IL-17, and tumor necrosis factor-alpha (TNF-α), although other markers are also currently under investigation and gradually being incorporated into this inflammatory profile (Su *et al.* 2018, Hu *et al.* 2020, Rostamtabar *et al.* 2021). This unfavorable immune profile compromises endometrial receptivity and decidualization, impairing maternal–fetal tolerance and favoring embryonic rejection. Furthermore, the hyperactivation of NK cells and the increase in inflammatory mediators in the uterine environment contribute to placental dysfunction and the elevated risk of pregnancy loss and other obstetric complications in this population (Bahri Khomami *et al.* 2024, Chen *et al.* 2024). This immune and inflammatory dysregulation, in association with MetS, obesity, hypertension, and IR, generates a self-perpetuating cycle of dysfunction, contributing to the development of a progressively deleterious condition, if not addressed through its various etiopathogenic pathways (Zhai *et al.* 2022).

PCOS is a clinically heterogeneous condition that can be classified into different phenotypes based on the Rotterdam criteria. The four main phenotypes result from different combinations of three diagnostic features: chronic anovulation, clinical or biochemical hyperandrogenism, and polycystic ovarian morphology. Phenotype A (the classic form) includes all three features and is typically associated with more severe manifestations, such as increased insulin resistance and higher cardiometabolic risk. Phenotypes B, C, and D represent various combinations of these criteria, each with distinct reproductive and metabolic profiles (Chan *et al.* 2024). This phenotypic diversity highlights the complexity of PCOS and underscores the need for individualized clinical approaches. Immune abnormalities can vary according to the different PCOS phenotypes. Hyperandrogenic phenotypes (A and B) present higher systemic inflammatory activation, with elevated levels of proinflammatory cytokines, insulin resistance, and immune dysfunction, which is associated with a higher risk of pregnancy loss and a lower pregnancy and live birth rate compared to non-hyperandrogenic phenotypes, even after adjusting for body mass index (BMI) (Chan *et al.* 2024).

This narrative review offers a unique contribution by specifically focusing on the immunological and inflammatory mechanisms underlying PCOS and their impact on reproductive outcomes – a dimension that remains poorly explored in the literature. Our work distinguishes itself by integrating current evidence on systemic and local immune dysregulation in reproductive tissues, highlighting how these immune alterations vary across PCOS phenotypes and contribute to ovulatory dysfunction, infertility, and pregnancy complications. By presenting this integrative and immunologically centered perspective, we aim to advance the understanding of PCOS pathophysiology and support the development of more targeted and personalized therapeutic strategies.

## Pathophysiology of PCOS and immune dysregulation

**Figure 1:**
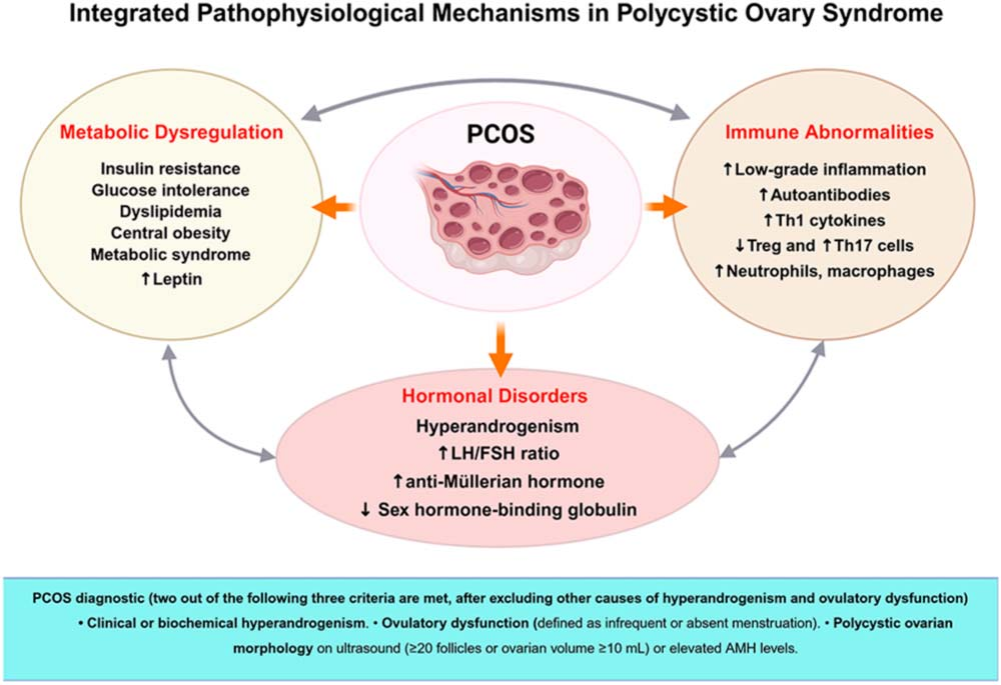
Integrated pathophysiological mechanisms in polycystic ovary syndrome. This figure illustrates the interconnected metabolic, hormonal, and immune abnormalities involved in the pathophysiology of PCOS. Metabolic dysregulation includes insulin resistance, glucose intolerance, dyslipidemia, central obesity, and elevated leptin levels. Hormonal disorders are characterized by hyperandrogenism, altered LH/FSH ratio, increased anti-Müllerian hormone (AMH), and decreased sex hormone-binding globulin (SHBG). Immune abnormalities involve chronic low-grade inflammation, increased Th1 cytokines, autoantibodies, and altered immune cell populations (e.g. Treg, Th17, neutrophils, and macrophages). These dysregulated systems interact and contribute to the development and persistence of PCOS. Diagnostic criteria are summarized below the diagram. Created in BioRender. https://BioRender.com/l4l22m0.

PCOS is a complex endocrine and metabolic condition characterized by chronic low-grade inflammation resulting from the interaction of hormonal, metabolic, and immunological dysfunctions. Although PCOS has traditionally been recognized for its reproductive manifestations – including anovulation and hyperandrogenism – and metabolic features – including IR and obesity – recent evidence has indicated that PCOS should also be understood as a systemic immunoinflammatory disorder, with direct repercussions on the ovarian and endometrial microenvironments, as well as broader systemic impacts (Rudnicka *et al.* 2021, Zhai *et al.* 2022) (Fig. 1).

From an endocrine perspective, hyperandrogenism, IR, and visceral obesity form a feedback loop that perpetuates immunometabolic imbalance. Insulin and androgens, by promoting lipogenesis and adipose tissue accumulation, contribute to the release of proinflammatory cytokines – including IL-6, TNF-α, and C-reactive protein (CRP) – by macrophages and adipocytes (Dumesic *et al.* 2015, Azziz *et al.* 2016, Zhai *et al.* 2022). These mediators activate the nuclear factor kappa B and NOD-like receptor family pyrin domain-containing 3 (NLRP3) inflammasome pathways, resulting in oxidative stress, granulosa cell apoptosis, and impaired steroidogenesis (González 2012, Escobar-Morreale 2018). This inflammatory environment not only compromises follicular development and oocyte maturation but also impairs endometrial receptivity and pregnancy outcomes (Orisaka *et al.* 2023, Dai *et al.* 2024) (Table 1).

**Table 1:** Summary of key inflammatory cytokines in PCOS: sources, targets, and clinical correlations.

Cytokine	Cellular sources	Primary targets	Clinical correlations in PCOS
IL-1β	Macrophages and granulosa cells	Ovarian stromal cells and endometrium	Follicular dysfunction and impaired steroidogenesis
IL-6	Macrophages, adipocytes, and endometrial cells	Granulosa cells, liver, and endothelium	Insulin resistance, reduced aromatase activity, and systemic inflammation
TNF-α	M1 macrophages and NK cells	Ovarian and endometrial cells	Anovulation, granulosa cell apoptosis, and endometrial dysfunction
IL-17	Th17 lymphocytes	Immune cells and endometrium	Inflammation, impaired endometrial receptivity, and miscarriage
IL-18	Macrophages and epithelial cells	Endothelial and immune cells	Oxidative stress and pregnancy complications
IFN-γ	Th1 and NK cells	Trophoblast and immune cells	Endometrial inflammation, maternal–fetal intolerance, and implantation failure

The inflammatory cytokines listed play central roles in the immunopathology of PCOS. IL-1β and TNF-α, produced by macrophages and granulosa cells, promote granulosa cell apoptosis and impair steroidogenesis (González *et al.* 2020, Liu *et al.* 2021*b*, Dai *et al.* 2024). IL-6, secreted by macrophages and adipocytes, is linked to insulin resistance and reduced aromatase activity (Hu *et al.* 2020, Zhai *et al.* 2022). IL-17 and IFN-γ, derived from Th17 and Th1 cells, respectively, disrupt maternal–fetal immune tolerance and endometrial receptivity, contributing to miscarriage and implantation failure (Krishna *et al.* 2015, Qin *et al.* 2016). IL-18, elevated in PCOS, promotes oxidative stress and adverse pregnancy outcomes (Rostamtabar *et al.* 2021, Bahri Khomami *et al.* 2024).

Histological and molecular studies have demonstrated a marked increase in macrophage and lymphocyte infiltration within the ovarian tissues of women with PCOS, characterized by a predominance of the proinflammatory M1 phenotype over the anti-inflammatory M2 profile (Lima *et al.* 2018, Zhai *et al.* 2022). The predominance of the M1 phenotype leads to the increased production of TNF-α, IL-1β, and IL-6, amplifying IR and perpetuating hyperandrogenism, establishing a vicious cycle between inflammation and hormonal dysfunction (González *et al.* 2020, Chen *et al.* 2024, Deng *et al.* 2024). Additionally, IL-6 reduces aromatase activity in granulosa cells, thereby inhibiting the conversion of androstenedione into estradiol and contributing to the androgenic environment characteristic of the syndrome (González *et al.* 2020, Chen *et al.* 2024).

Low-grade systemic inflammation observed in PCOS is further supported by elevated serum levels of several markers, including IL-18, monocyte chemoattractant protein-1 (MCP-1), and interferon-gamma (IFN-γ), which correlate with obesity severity and IR (Rostamtabar *et al.* 2021, Rudnicka *et al.* 2021). This inflammatory condition is also evident in the follicular fluid, where increased IL-6, TNF-α, and IL-1β levels, alongside decreased levels of IL-10 – an anti-inflammatory cytokine – have been observed (Zhai *et al.* 2022, Banerjee *et al.* 2025). The combined effects of proinflammatory cytokines and oxidative stress induce lipid peroxidation and mitochondrial dysfunction, ultimately impairing oocyte competence and reducing embryo quality (Voros *et al.* 2025) (Table 1).

**Figure 2:**
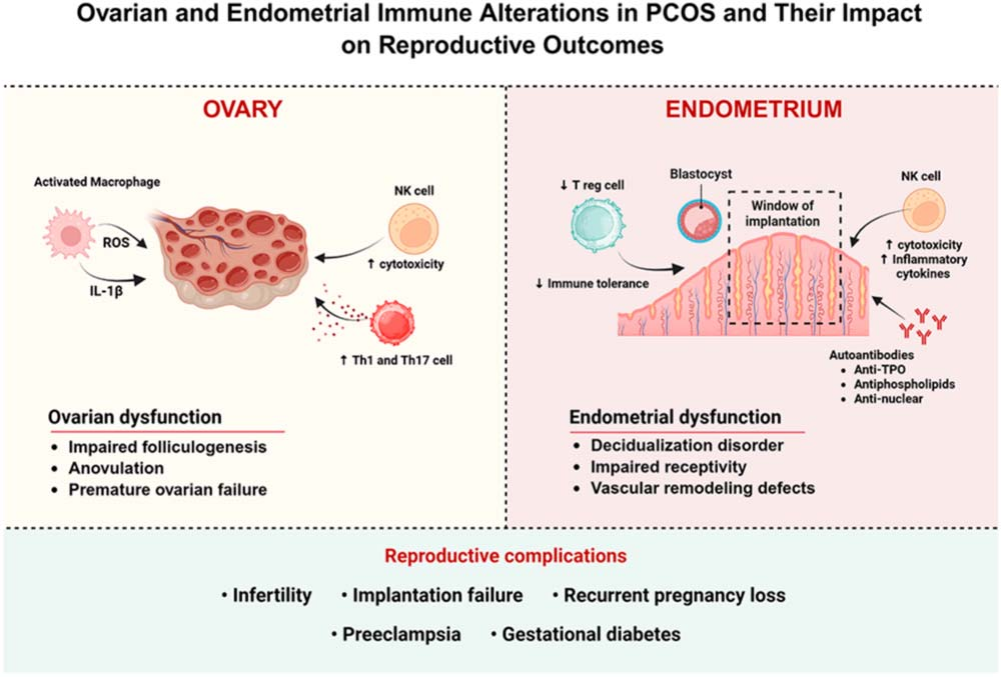
Ovarian and endometrial immune alterations in PCOS and their impact on reproductive outcomes. This figure illustrates the immunological alterations in the ovary and endometrium that contribute to reproductive complications in women with polycystic ovary syndrome (PCOS). In the ovary, activated macrophages produce reactive oxygen species (ROS) and IL-1β, while increased Th1/Th17 cells and cytotoxic NK cells contribute to impaired folliculogenesis, anovulation, and premature ovarian failure. In the endometrium, reduced T regulatory cells impair immune tolerance, and elevated inflammatory cytokines and NK cell cytotoxicity disrupt the window of implantation. The presence of autoantibodies (anti-TPO, antiphospholipid, and anti-nuclear) further compromises endometrial receptivity and vascular remodeling. Together, these immune-mediated dysfunctions are associated with adverse reproductive outcomes, including infertility, implantation failure, recurrent pregnancy loss, preeclampsia, and gestational diabetes. Created in BioRender. https://BioRender.com/4wj0t3c.

Regarding adaptive immunity, an imbalance between Th1/Th2 and Th17/Treg lymphocytes has been observed, with a predominance of Th1 and Th17 responses responsible for the secretion of IFN-γ and IL-17, which promote macrophage activation and tissue infiltration (Krishna *et al.* 2015, Qin *et al.* 2016, Zhai *et al.* 2022). The number and functional activity of Treg cells, which are essential for controlling inflammation and maintaining immune tolerance, are reduced in patients with PCOS, contributing to a hostile, proinflammatory ovarian microenvironment (Krishna *et al.* 2015). This dysfunction in adaptive immunity appears to be associated with poorer reproductive outcomes, including implantation failure and early pregnancy loss (Velez *et al.* 2021) (Fig. 2).

Another key component in the immunological pathophysiology of PCOS is gut dysbiosis. In healthy women, the gut microbiota plays a crucial role in modulating the immune response, promoting immune tolerance and intestinal barrier integrity, and producing anti-inflammatory metabolites such as short-chain fatty acids (SCFAs). Women with PCOS tend to have intestinal dysbiosis, characterized by reduced microbial diversity and specific alterations in bacterial composition. These include an increase in *Bacteroides, Parabacteroides, Escherichia/Shigella,* and *Lactobacillus* and a decrease in *Lachnospira*, *Prevotella*, and *Faecalibacterium prausnitzii*, with the latter three being producers of SCFAs and anti-inflammatory modulators (Chu *et al.* 2020, Li *et al.* 2023*a*, Hanna *et al.* 2025). This gut dysbiosis leads to increased intestinal permeability, facilitating the translocation of bacterial lipopolysaccharides (LPS) into the systemic circulation. LPS activate inflammatory pathways, consequently promoting the release of proinflammatory cytokines (IL-6, TNF-α, and MCP-1), activation of macrophages, and exacerbation of innate immune response. LPS also contribute to IR and hyperandrogenism (Pathak *et al.* 2025, Sharma *et al.* 2025). The reduction in SCFA-producing bacteria decreases anti-inflammatory signaling, thereby worsening the chronic low-grade inflammatory state typical of PCOS. Furthermore, alterations in bile acid metabolism and a reduction in IL-22 level – a cytokine that protects the intestinal mucosa – are also observed in women with PCOS, perpetuating immune and metabolic dysfunctions (Qi *et al.* 2019). The recently proposed gut–immune–ovary axis suggests that microbiota modulation through diet, prebiotics, or probiotics represents a promising therapeutic strategy to reduce systemic inflammation and restore reproductive function in women with PCOS (Pathak *et al.* 2025, Sharma *et al.* 2025).

Thus, the constellation of metabolic, endocrine, and immunological alterations observed in PCOS supports the concept of an immunoendocrine-based syndrome, in which metabolic, hormonal, and immune pathways interact in an integrated manner. Understanding these interconnections is essential for developing therapeutic strategies that not only address hyperandrogenism and IR but also attenuate chronic inflammation and restore immune homeostasis (Fig. 1).

## Autoimmunity and PCOS

Recently, the existing literature has increasingly supported the hypothesis that PCOS not only is an endocrine–metabolic disorder but also encompasses autoimmune components that contribute to its pathophysiology and influence women’s reproductive outcomes. The concurrent presence of low-grade chronic inflammation, immune dysfunction, and specific autoantibodies supports the notion that PCOS may represent a pro-autoimmune state, characterized by aberrant activation of the innate and adaptive immune systems and partial loss of immune tolerance (Kwiatkowski *et al.* 2025*b*).

Patients with PCOS have a markedly elevated risk of developing autoimmune diseases as compared to the general population. In a large population-based cohort, Glintborg *et al.* (2025) observed a 50% higher overall risk of autoimmune conditions in women with PCOS, corresponding to an incidence rate ratio (IRR) of 1.501 (95% confidence interval (CI) = 1.439–1.565; *P* < 0.001) (Glintborg *et al.* 2025). Among specific diseases, autoimmune thyroid disorders showed the strongest association (IRR = 1.48; 95% CI = 1.41–1.57; *P* < 0.001) (Glintborg *et al.* 2025). Recent systematic reviews have confirmed the elevated risk of Hashimoto’s thyroiditis in women with PCOS (odds ratio (OR) = 2.28; 95% CI = 1.61–3.22 and OR = 2.38; 95% CI = 1.63–3.49; Hu *et al.* 2022, Bahreiny *et al.* 2024). Beyond the thyroid autoimmunity, PCOS has been linked to an elevated risk of other immune-mediated conditions, including psoriasis (OR = 2.07; 95% CI = 1.25–3.43; *P* < 0.01), type 1 diabetes mellitus (IRR = 3.49; 95% CI = 3.17–3.87; *P* < 0.001), and broader categories of skin (IRR = 1.55; 95% CI = 1.32–1.81; *P* < 0.001), digestive (IRR = 1.55; 95% CI = 1.32–1.81; *P* < 0.001), and musculoskeletal autoimmune diseases (IRR = 1.50; 95% CI = 1.44–1.56; *P* < 0.001) (Lee *et al.* 2020*b*, Glintborg *et al.* 2025). Although rheumatoid arthritis exhibited only a nonsignificant trend toward higher prevalence (odds ratio (OR) = 1.81; *P* = 0.075), and multiple sclerosis did not show a significant association (OR = 0.90; *P* = 0.07), the overall pattern indicates a systemic predisposition to immune dysregulation among women with PCOS (Sharmeen *et al.* 2021, Jiang *et al.* 2024). These findings indicate that PCOS extends beyond an endocrine–metabolic disorder, encompassing a broader immunometabolic imbalance that increases susceptibility to multiple autoimmune conditions (Table 2).

**Table 2:** Risk of autoimmune diseases in women with polycystic ovary syndrome: summary of key studies and effect estimates.

Autoimmune disease	Risk of autoimmune disease in women with PCOS					Reference
	OR	IRR	95% CI	*I* ^2^	*P*	
Hashimoto’s thyroiditis	2.38		1.63–3.49	50.1%	<0.001	Bahreiny *et al.* (2024)
Hashimoto’s thyroiditis	2.28		1.61–3.22	63.1%	<0.0001	Hu *et al.* (2022)
Thyroid disorder		1.48	1.41–1.57		<0.001	Glintborg *et al.* (2025)
Rheumatoid arthritis	1.81		0.93–3.4		0.0747	Sharmeen *et al.* (2021)
Psoriasis	2.07		1.25–3.43		<0.01	Lee *et al.* (2020*a*,*b*)
Skin autoimmune disease		1.55	1.32–1.81		<0.001	Glintborg *et al.* (2025)
Type 1 diabetes mellitus		3.49	3.166–3.87		<0.001	Glintborg *et al.* (2025)
Multiple sclerosis	0.90		0.80–1.01		0.07	Jiang *et al.* (2024)
Digestive autoimmune disease		1.55	1.32–1.81		<0.001	Glintborg *et al.* (2025)
Musculoskeletal autoimmune disease		1.50	1.44–1.56		<0.001	Glintborg *et al.* (2025)

OR, odds ratio. IRR: incidence rate ratio. Thyroid disorder was defined as thyrotoxicosis, autoimmune thyroiditis, and/or prescription of thyroid. Skin autoimmune disease includes the following diseases: pemphigus, pemphigoid, dermatitis herpetiformis, psoriasis vulgaris, erythema nodosum, alopecia areata, vitiligo, lupus erythematosus, and lichen sclerosus. Digestive autoimmune diseases include the following: Crohn’s disease, ulcerative colitis, autoimmune hepatitis, primary biliary cirrhosis, and celiac disease; musculoskeletal autoimmune diseases include the following: Reiter’s disease, seropositive rheumatoid arthritis, juvenile arthritis, polyarteritis nodosa, Kawasaki syndrome, Goodpasture’s syndrome, Wegener’s granulomatosis, polymyalgia rheumatica, systemic lupus erythematosus, dermatopolymyositis, systemic sclerosis, Sjogren’s syndrome, Behcet’s disease, Ankylosing spondylitis, and Dupuytren’s disease.

The imbalance between the Th1/Th2 and Th17/Treg lymphocyte subtypes is among the main mechanisms linking autoimmunity and PCOS (Krishna *et al.* 2015, Qin *et al.* 2016, Kwiatkowski *et al.* 2025*b*). The predominance of the Th1 response, characterized by the production of IFN-γ and tumor TNF-α, promotes macrophage recruitment and amplifies tissue inflammation, directly affecting the ovarian microenvironment (Qin *et al.* 2016, Su *et al.* 2018, Zhai *et al.* 2022). Concurrently, the increase in Th17 cells and IL-17 levels is associated with follicular dysfunction, granulosa cell apoptosis, and endometrial receptivity alterations (Dai *et al.* 2024). Conversely, the reduction in Treg cells, which produce IL-10 and transforming growth factor-beta (TGF-β), impairs peripheral tolerance mechanisms and may contribute to both autoimmunity and recurrent pregnancy loss (RPL) (Krishna *et al.* 2015) (Fig. 2).

Hormonal mechanisms play an important modulatory role in this context. Estrogen, at physiological concentrations, exerts immunoprotective and anti-inflammatory effects; however, when present in excess, it can stimulate the proliferation of autoreactive B lymphocytes and increase autoantibody production (Cutolo *et al.* 2006). Progesterone and glucocorticoids tend to promote immune tolerance; however, the resistance to these hormones – commonly observed in women with PCOS – favors a proinflammatory polarization (Da Silva 1999, Luan *et al.* 2022). Additionally, hyperandrogenism – although classically associated with ovulatory dysfunction – also modulates an immune response, as androgens reduce Treg cell differentiation and enhance the cytotoxic activity of NK cells, thereby perpetuating the inflammatory state (Wang *et al.* 2023). The activation of the NLRP3 inflammasome pathway and IL-1β/IL-18 axis has been identified in both the ovaries and follicular fluid of patients with PCOS and positive autoantibodies, suggesting that sterile inflammation triggers a local autoimmune phenomenon (Liu *et al.* 2021*b*, Wang *et al.* 2024). The consequent release of these proinflammatory mediators induces granulosa cell apoptosis, promotes ovarian fibrosis, and impairs steroidogenesis, thereby exacerbating hormonal imbalance (Liu *et al.* 2021*b*, Xiang *et al.* 2023).

In the reproductive setting, autoimmune abnormalities and immune dysregulation in women with PCOS have been increasingly associated with adverse gestational outcomes, particularly implantation failure and RPL (Cavalcante *et al.* 2018). The coexistence of autoimmune diseases or subclinical immune activation may disrupt the delicate balance required for embryo implantation and maternal–fetal tolerance. Elevated levels of autoantibodies, including antinuclear, antithyroid, and antiphospholipid antibodies, are consistently associated with adverse reproductive outcomes (Cavalcante *et al.* 2020, Fang *et al.* 2022, Cavalcante *et al.* 2025*c*). These autoantibodies may impair embryo implantation by promoting a proinflammatory uterine microenvironment, altering endometrial receptivity, and interfering with trophoblast invasion and vascular remodeling, thereby linking autoimmune activation to poor gestational outcomes in women with PCOS and other reproductive disorders (Fig. 2). These findings emphasize that the immune dysfunction observed in PCOS is systemic and uterine in nature, directly affecting the endometrium–embryo interface and contributing to reproductive failures. Therefore, the investigation of autoimmune abnormalities – including thyroid autoimmunity and antiphospholipid antibodies – is essential in the evaluation of women with reproductive disorders, particularly those with PCOS, to guide individualized management and improve pregnancy outcomes (Kwak-Kim *et al.* 2022, Kwiatkowski *et al.* 2025*a*).

Therefore, understanding PCOS through the perspective of immunoregulation and autoimmunity broadens the traditional paradigm centered solely on endocrinopathy. The identification of phenotypic subgroups with greater autoimmune predisposition, as well as the recognition of inflammatory and immunological biomarkers, may guide the future development of personalized therapeutic strategies that integrate immune modulation, metabolic control, and hormonal management as key pillars in PCOS treatment.

## Endometrial immune environment in PCOS

Endometrial receptivity refers to the ability of the endometrium to enable embryo adhesion and implantation during the ‘window of implantation’. It comprises a set of morphological, molecular, and functional modifications occurring in the endometrium to enable successful embryo implantation and support early embryonic development (Norwitz *et al.* 2001, Paulson 2019). This process is mainly regulated by ovarian steroids, particularly estrogen, which promotes endometrial tissue proliferation, and progesterone, which induces secretory differentiation, thereby functionally preparing the endometrium for embryo implantation, establishing an optimal period for embryo localization, adhesion, trophoblast invasion, and implantation (Norwitz *et al.* 2001, Paulson 2019). Synchronization between the embryonic developmental stage and endometrial maturation degree is essential, as any disruption in this process may compromise the success of implantation (Norwitz *et al.* 2001, Paulson 2019).

Morphological markers of endometrial receptivity during the implantation window include pinopodes, which are cytoplasmic projections that develop on the apical surface of the endometrial epithelium (Quinn *et al.* 2020). These structures result from the fusion and transformation of microvilli into broad extensions that cover the apical membrane (Quinn *et al.* 2020). Pinopodes are essential for facilitating blastocyst adhesion and regulating the luminal environment through the absorption of fluid and signaling molecules. The analysis of pinopode density and maturation serves as a morphological biomarker for determining endometrial receptivity status (Quinn *et al.* 2020, Zhu *et al.* 2025).

Women with PCOS present considerable alterations in the endometrium during the implantation window, particularly reduced pinopode maturation. This structural immaturity impairs endometrial receptivity (Palomba *et al.* 2021). PCOS is commonly associated with hyperandrogenism, which is characterized by excessive androgen production and reduced conversion to estrogen due to decreased aromatase activity. This hormonal imbalance, compounded by IR and low sex hormone-binding globulin levels, increases free testosterone concentrations and induces marked endometrial alterations (Lee *et al.* 2020*a*, Wei *et al.* 2024).

Hyperandrogenism interferes with the expression of key genes and proteins involved in receptivity, including HOXA10, HOXA11, and integrin αVβ3, as well as critical signaling pathways including leukemia inhibitory factor, CDK, MECA-79, and MAGEA-11, which are essential for implantation (Palomba *et al.* 2021, Guo *et al.* 2022, Parker *et al.* 2025). These alterations impair decidualization by disrupting stromal cell differentiation, thereby reducing the capacity for embryo adhesion, invasion, and implantation, ultimately leading to decreased fertility rates (Palomba *et al.* 2021, Guo *et al.* 2022, Parker *et al.* 2025). Furthermore, hyperandrogenism disrupts intercellular junctions, decreasing their complexity and downregulating claudin-4 and occludin expressions, thereby compromising the endometrial barrier integrity and embryonic adhesion (Matsuyama *et al.* 2024, Parker *et al.* 2025). The combined effects of hormonal, metabolic, and molecular factors induced by PCOS establish a less receptive endometrial environment, accounting for the adverse reproductive outcomes frequently observed in PCOS.

Many immune cells express estrogen and progesterone receptors, and their functions are directly modulated by hormonal fluctuations (Brundin *et al.* 2021). Consequently, the female immune environment exhibits a unique dynamic, wherein hormonal variations influence the production of cytokines and chemokines as well as the distribution of immune cell populations. During the follicular phase, when estrogen predominates, the immune profile is predominantly modulatory and anti-inflammatory. High estrogen levels suppress proinflammatory cytokines, including IL-1β and TNF-α, while promoting the release of immunoregulatory mediators, including IL-4, IL-10, and TGF-β. This regulation contributes to a less aggressive immune response, protecting tissues from excessive inflammation (O’Brien *et al.* 2007, Weinberg *et al.* 2011). At high concentrations, progesterone similarly exerts immunoregulatory effects, reinforcing Th2 polarization and promoting immune tolerance, which are particularly critical during pregnancy (Raghupathy & Szekeres-Bartho 2022). However, this action does not preclude the emergence of a proinflammatory immune environment during specific phases, including the luteal phase. This reflects the adaptive and dynamic nature of the interaction between sex hormones and the immune system. In the luteal phase, the levels of proinflammatory cytokines, including TNF-α, IL-1β, and IL-6, increase significantly, playing a key role in endometrial preparation for embryo implantation (Raghupathy & Szekeres-Bartho 2022) (Table 1). These cytokines facilitate tissue remodeling and influence the migration and activity of immune cells, such as monocytes and neutrophils, which actively participate in endometrial adaptation. Additionally, immune cell phenotypes become more responsive to inflammatory stimuli, highlighting the importance of controlled inflammation for implantation (Arck *et al.* 2007, Raghupathy & Szekeres-Bartho 2022).

Women diagnosed with PCOS exhibit a state of low-grade chronic inflammation, accompanied by increased endometrial infiltration of immune cells, including macrophages (CD68+ and CD163+), dendritic cells (CD1a+ and CD83+), CD8+ T lymphocytes, and CD56+ NK cells (Liu *et al.* 2021*a*). This inflammatory profile manifests independently of BMI, suggesting that it is intrinsic to PCOS and not merely a consequence of obesity. The imbalance in proinflammatory cytokine expressions (IL-6, IL-8, TNF-α, MCP-1, and GM-CSF) in epithelial and stromal cells, as well as the increase in adhesion molecules and metalloproteinases, reportedly favors the recruitment and migration of immune cells to the endometrium (Piltonen *et al.* 2015, Palomba *et al.* 2021). The endometrial microenvironment exhibits progesterone resistance, thereby impairing decidualization and immunological tolerance, which, in turn, contributes to implantation failures and an increased risk of gestational complications. Furthermore, there is altered insulin signaling and increased local cortisol, exacerbating endometrial IR and perpetuating the inflammatory state. The molecular profile reveals the activation of pathways, such as PI3K/AKT/MAPK and Wnt/β-catenin, associated with inflammation and cell proliferation (Parker *et al.* 2025).

Uterine natural killer (uNK) cells play a central role in early placentation by regulating extravillous trophoblast (EVT) invasion and remodeling of the decidual spiral arteries (Díaz-Hernández *et al.* 2021). These cells are highly abundant in the first trimester and secrete various cytokines and growth factors, ensuring that trophoblast invasion is controlled. The uNK cells are, therefore, key elements in implantation and early functional placental development by facilitating proper spiral artery remodeling (Díaz-Hernández *et al.* 2021, Xu *et al.* 2022). The uNK cells initiate decidualization by inducing early structural changes in spiral arteries, such as loosening of the medial layer. As the implantation progresses, EVTs invade the uterine vessels and induce smooth muscle cell degeneration, which is gradually replaced by fibrinoid materials (Díaz-Hernández *et al.* 2021).

Previous studies have indicated a substantial decline in the proportion of endometrial NK cells, particularly the CD56bright/CD16-subtype, during the secretory phase of the menstrual cycle in women diagnosed with PCOS, in comparison to fertile women without PCOS. Furthermore, there is an observed decrease in the expression of cytokines that are essential for the recruitment and maturation of these cells, including IL-15 and IL-18 (Matteo *et al.* 2010). This condition contributes to an endometrial microenvironment that is less receptive to embryonic implantation. These alterations are not influenced by BMI, thereby indicating that they are intrinsic to the PCOS pathophysiology. The reduction in the number of endometrial NK cells is associated with an impaired cytokine network, potentially accounting for the decreased reproductive potential, implantation failures, and increased pregnancy complication risk observed in women with PCOS (Palomba *et al.* 2021). Immunological changes in the endometrium of women diagnosed with PCOS are directly related to subfertility, implantation failure, recurrent miscarriage, and an increased risk of endometrial hyperplasia and cancer.

## Reproductive outcomes among patients with PCOS

Women with PCOS are at increased risk of adverse reproductive and obstetric outcomes, such as infertility, recurrent pregnancy loss, gestational diabetes mellitus, gestational hypertension, preeclampsia, and preterm delivery. These unfavorable outcomes result from a complex network of interactions among chronic low-grade inflammation, IR, hyperandrogenism, and endometrial dysfunction – reflecting the integration of immunological, metabolic, and endocrine pathways in the pathophysiology of the syndrome (McCartney & Marshall 2016). Given the clinical heterogeneity of PCOS, characterized by distinct phenotypes based on combinations of anovulation, hyperandrogenism, and polycystic ovarian morphology, it is essential to consider these differences when evaluating reproductive outcomes and guiding individualized management strategies. Hyperandrogenic phenotypes, in particular, are associated with greater inflammatory activation and worse reproductive prognoses (Chan *et al.* 2024).

From an endocrine–metabolic perspective, IR, hyperandrogenism, and related metabolic disturbances commonly observed in PCOS contribute to impaired oocyte quality, altered folliculogenesis, and suboptimal endometrial receptivity. However, growing evidence has shown that immune dysregulation in women with PCOS contributes to poor gestational outcomes. Immunological alterations in the uterine environment, systemic immune changes, increased risk of autoimmune abnormalities, and disorders in the innate immune response justify the worsening of reproductive performance among women with PCOS (McCartney & Marshall 2016).

The chronic proinflammatory processes in women with PCOS contribute to the development of poor-quality oocytes, thereby promoting anovulatory infertility and accelerating ovarian reserve depletion (Xiong *et al.* 2011; Boots & Jungheim 2015). A cohort study evaluating patients with PCOS undergoing assisted reproductive technology (ART), particularly *in vitro* fertilization (IVF) or intracytoplasmic sperm injection (ICSI), revealed that the adverse inflammatory and metabolic profile inherent to patients with PCOS considerably affects the pregnancy and neonatal outcomes (Du & Ge 2025). The study demonstrated that the PCOS-IVF/ICSI group exhibited a markedly heightened inflammatory response compared with women with spontaneous pregnancies, with elevated levels of inflammatory markers throughout the early, mid, and late stages of pregnancy. These markers included CRP and ILs, including IL-2, IL-4, IL-6, IL-8, IL-12, and IL-18 (Du & Ge 2025). This exacerbated inflammation, often associated with progressive IR, appears to be a key factor contributing to the increased risk of gestational complications (Armanini *et al.* 2022, Li *et al.* 2023*b*). Consequently, patients with PCOS who underwent IVF/ICSI had markedly higher rates of pregnancy complications, including spontaneous miscarriage and gestational hypertension. Moreover, their neonates presented considerably lower birth weights and reduced Apgar scores (both at 1 and 5 min), indicating worse early neonatal outcomes. These findings suggest that individualized management of metabolic and inflammatory status helps reduce the risk of developing complications and improve maternal and neonatal outcomes following IVF/ICSI in women with PCOS (Du & Ge 2025).

A comprehensive meta-analysis encompassing 104 studies and over 106,000 pregnancies confirmed that women with PCOS have a considerably higher likelihood of developing pregnancy complications. Increased risks of adverse outcomes, including spontaneous miscarriage (OR = 1.49; 95% CI = 1.20–1.85), gestational diabetes mellitus (OR = 2.41; 95% CI = 1.95–2.99), gestational hypertension (OR = 2.20; 95% CI = 1.81–2.69), preeclampsia (OR = 2.30; 95% CI = 1.87–2.82), and cesarean delivery (OR = 1.23; 95% CI = 1.06–1.43) were observed. These increased risks are likely to be independent of maternal age and BMI, as the associations remained significant even in sensitivity analyses using age- or BMI-matched controls (Bahri Khomami *et al.* 2024).

The role of low-grade chronic inflammation in PCOS development and progression is now well established, with implications for the clinical manifestations and metabolic and reproductive outcomes of the syndrome (Velez *et al.* 2021). In this context, emerging inflammatory biomarkers aim to more accurately and integratively reflect the systemic immune status, enabling not only prognostic stratification but also personalized therapeutic targeting. One such marker is the Systemic Immune–Inflammation Index (SII) – a low-cost parameter calculated using the ratio of platelet and neutrophil counts to lymphocyte counts – which has already been explored in obstetric settings (Tanacan *et al.* 2020). Recently, its application in patients with PCOS undergoing IVF has demonstrated that elevated SII levels are associated with poorer reproductive outcomes. Specifically, women in the highest SII quartile exhibited a reduced number of viable embryos and blastocyst formation rates. Multivariate analysis further indicated significantly lower rates of implantation, clinical or biochemical pregnancy, and live birth in this group. These findings suggest that SII reflects a systemic immune activation state that negatively affects the endometrial environment and impairs implantation and placentation, highlighting its potential as a promising prognostic biomarker and tool for ART strategy optimization among patients with PCOS (Li *et al.* 2023*b*).

Women with PCOS are 49–53% more likely to experience miscarriage, and recurrent pregnancy loss has also been identified as a pattern in this population (Wartena *et al.* 2023, Bahri Khomami *et al.* 2024). The key biomarkers associated with this correlation include elevated levels of homocysteine, CRP, C1q/TNF-alpha-related protein 6 (CTRP6), and plasma leptin; reduced insulin sensitivity; and decreased adiponectin levels (Moini *et al.* 2012, Sadeghi *et al.* 2020, Kargasheh *et al.* 2021). Coagulation factors, such as increased plasminogen activator inhibitor activity that is indicative of hypofibrinolysis, have been identified as independent predictors of adverse pregnancy outcomes in patients with PCOS, with a higher frequency of thrombophilic disorders also observed (Glueck *et al.* 2006, Cavalcante *et al.* 2019). Regarding the hormonal and phenotypic factors, hyperandrogenism has been linked to a higher relative risk of miscarriage, and an elevated serum AMH level (>6.1 ng/mL) is significantly associated with an increased risk of pregnancy loss (Wunder *et al.* 2008).

Therefore, immune dysfunction represents a key mechanistic link between inflammation and adverse reproductive outcomes in PCOS. This relationship is driven by an increased prevalence of autoimmune diseases and functional imbalances in maternal immune regulation, including reduced regulatory T cells, expansion of Th1/Th17 responses, heightened NK cell activity, and excessive production of proinflammatory cytokines. These immune alterations compromise maternal–fetal tolerance; impair implantation, decidualization, and uteroplacental vascular remodeling; and promote placental dysfunction. In parallel, cytokine dysregulation and autoantibody-mediated mechanisms contribute to endothelial injury, thrombosis, and metabolic disturbances, ultimately increasing the risk of pregnancy loss, gestational diabetes, hypertensive disorders of pregnancy, preeclampsia, and preterm birth in women with PCOS (Fig. 2).

## Immunomodulatory therapies and emerging treatments

Treatments for PCOS can be categorized into the following two distinct approaches: nonpharmacological and pharmacological interventions. Lifestyle modifications that include an enhanced diet, greater physical activity, improved stress management, and better sleep quality are fundamental for improving reproductive outcomes. Major international guidelines recommend that lifestyle interventions should be the primary management option for all women with PCOS (Teede *et al.* 2023). Such interventions should focus on weight gain prevention, fertility optimization, and metabolic and cardiovascular risk reduction.

Previous studies have demonstrated an association between an inflammatory diet and suboptimal obstetric and perinatal outcomes (de Freitas *et al.* 2022, Chagas *et al.* 2025). Therefore, dietary interventions incorporating foods with a lower inflammatory index, with or without weight reduction, improve pregnancy outcomes. These interventions have been shown to reduce systemic inflammatory markers, including IL-6, TNF-α, and CRP, improve ovarian and uterine immune response, and favor the endometrial microenvironment, leading to better implantation and pregnancy outcomes (de Freitas *et al.* 2022).

In women diagnosed with PCOS, physical activity has a beneficial effect on hormonal, metabolic, and immune modulation, thereby reducing both the systemic and local inflammatory states (Parmar & Whooten 2024, Cavalcante *et al.* 2025*a*). Regular exercise decreases proinflammatory cytokine levels, thereby contributing to reduced endometrial inflammation and enhanced uterine receptivity. Increased insulin sensitivity reduces the activation of inflammatory pathways associated with IR. Furthermore, an improvement in the immune cell profile, including the normalization of uNK cell function and a reduction in activated macrophage infiltration, has been observed (Butt *et al.* 2023). Moderate-to-vigorous-intensity exercise, particularly when performed for a minimum duration of 120 min per week, considerably reduces inflammation and enhances immunological markers (Patten *et al.* 2020).

Metformin is a widely used drug for treating PCOS, especially in the presence of IR. The main immune mechanisms of metformin involve the modulation of the differentiation and activation of various immune-mediated cells, including CD4+ and CD8+ T cells, and the reprogramming of intracellular metabolic pathways. Metformin exerts its immune effects by regulating important metabolic and immunological signaling pathways. Among the immunological effects of metformin, the inhibition of Th1 and Th17 cell differentiation and promotion of the development and increase of Treg cells contribute to improved reproductive outcomes in women with PCOS (Nojima & Wada 2023) (Table 3).

**Table 3:** Therapeutic agents with potential immunomodulatory effects in PCOS.

Therapeutic agent	Immunological mechanisms	Reproductive impact	Evidence level
Metformin	Promotes Treg and inhibits Th17 and Th1	Improves ovulation and implantation	High (RCTs and meta-analyses)
GLP-1RAs	Reduce proinflammatory cytokines and enhance Treg and M2 macrophages	Improve implantation and endometrial receptivity	Moderate (clinical studies and mechanistic data)
Vitamin D	Enhances Treg function and reduces Th1/Th17	Supports immune tolerance and endometrial receptivity	Moderate (observational studies and trials in progress)
Corticosteroids	Suppress Th1/Th17 and reduce NK activity	May benefit autoimmune subtypes	Low–moderate (limited studies in PCOS)
IVIG/lipid emulsion	Reduces NK cytotoxicity and balances cytokines	Improves implantation in select immune-mediated cases	Low (mainly ART context)
Calcineurin inhibitors	T-cell modulation and immune suppression	Potential benefit in immune-mediated infertility	Low (case series)
Hydroxychloroquine	Anti-inflammatory; reduces autoantibody production	May improve outcomes in APS/thyroid autoimmunity	Low (off-label, limited data)

GLP-1RAs, GLP-1 receptor agonists; IVIG, intravenous immunoglobulin; calcineurin inhibitors: tacrolimus/cyclosporine. The immunomodulatory effects of therapeutic agents in PCOS have been increasingly investigated (Cavalcante *et al.* 2025*a*). Metformin promotes regulatory T cells (Treg) and suppresses Th1/Th17 responses, contributing to improved ovulation and implantation (Nojima & Wada 2023). GLP-1 receptor agonists modulate systemic inflammation and enhance endometrial immune balance by increasing Treg and M2 macrophage polarization (Tavares *et al.* 2025). Vitamin D plays a role in immune tolerance by enhancing Treg activity and reducing Th1/Th17 cytokines, with promising effects on endometrial receptivity (Cavalcante *et al.* 2025*a*). Corticosteroids may benefit selected patients with autoimmune features by suppressing proinflammatory immune cells and NK activity (Cavalcante *et al.* 2025*a*). IVIG and lipid emulsions have shown potential in reducing NK cell cytotoxicity and regulating cytokine profiles, particularly in assisted reproduction contexts (Cavalcante *et al.* 2025*a*). Calcineurin inhibitors (e.g. tacrolimus and cyclosporine) modulate T cell activation and have been used in immune-mediated infertility (Nakagawa *et al.* 2017, Cavalcante *et al.* 2025*a*). Hydroxychloroquine, though off-label in PCOS, may improve outcomes in cases with antiphospholipid syndrome or autoimmune thyroid disease by reducing autoantibody production and systemic inflammation (Cavalcante *et al.* 2025*a*).

Glucagon-like peptide-1 receptor agonists (GLP-1RAs), initially developed for the treatment of type 2 diabetes mellitus and obesity, have shown therapeutic potential in obesity-associated PCOS owing to their metabolic, vascular, and immunological effects (Hudanich *et al.* 2025, Piazza 2025). Several immunological effects of GLP-1RAs have already been described. The main immunological mechanism by which GLP-1RAs may improve the outcomes is mediated by increased frequency and functionality of Treg cells and restoring the Th1/Th2 and Th17/Treg balance, which is frequently altered in reproductive disorders (Table 3). Furthermore, GLP-1RAs reduce the production of inflammatory cytokines, including IFN-γ, IL-4, IL-8, and IL-13, and modulate macrophage polarization, favoring the anti-inflammatory M2 phenotype and suppressing the proinflammatory M1 phenotype. They also contribute to the normalization of NK cell activity by reducing their cytotoxic activity (Tavares *et al.* 2025). In addition to the metabolic and vascular effects, these agents improve vascularization and endothelial function and reduce oxidative stress at the maternal–fetal interface, creating a more favorable environment for implantation and maintenance of pregnancy. Despite their promising potential in preconception protocols, their use during pregnancy remains contraindicated due to a lack of evidence of fetal safety (Tavares *et al.* 2025).

Although PCOS is primarily a metabolic–endocrine disorder, it is also characterized by immune dysregulation that contributes to poorer reproductive outcomes. Currently, immunotherapies are not recommended as a routine treatment for PCOS-related reproductive dysfunctions, as improvements in immune profiles usually occur indirectly through conventional interventions, such as lifestyle modification, insulin sensitizer use, and hormonal regulation (Teede *et al.* 2023). However, emerging evidence suggests potential future applications of targeted immunomodulation in selected cases with immune abnormalities (Cavalcante *et al.* 2025*b*).

Corticosteroids could help restore the Th1/Th2 balance and reduce uNK cell activity, potentially benefiting PCOS women with autoimmune markers, such as elevated antinuclear antibodies or uNK cell expansion (Cavalcante *et al.* 2025*b*). Intravenous immunoglobulin and lipid emulsions may downregulate NK cell cytotoxicity and normalize Th1 cytokine dominance, improving implantation in cases of immune-mediated reproductive failure. Vitamin D stands out as a safe immunoregulatory option, reducing Th1/Th17 activation while enhancing Treg cell function and endometrial receptivity. Calcineurin inhibitors (tacrolimus or cyclosporine) and hydroxychloroquine could also modulate excessive immune activation in selected autoimmune phenotypes, with the latter being particularly useful in the presence of antiphospholipid or antithyroid antibodies. Additionally, G-CSF and anti-TNF agents might rebalance the Th1/Th17 and Treg pathways and enhance decidualization, whereas hCG supports local immune tolerance and angiogenesis, although with limited evidence (Cavalcante *et al.* 2025*b*) (Table 3).

In summary, although immunological mechanisms are increasingly recognized in the pathophysiology of PCOS, substantial knowledge gaps still limit their clinical application. A major challenge is the identification and validation of immune biomarkers capable of reliably predicting adverse reproductive outcomes, which would enable patient stratification and support personalized therapeutic strategies. Moreover, there is a pressing need for well-designed, large-scale randomized clinical trials to evaluate the efficacy and safety of targeted immunomodulatory therapies aimed at improving fertility and obstetric outcomes in this population. Current evidence is constrained by methodological heterogeneity, small sample sizes, and inconsistencies in diagnostic criteria, which contribute to conflicting results and limit clinical translation. At present, immune-based therapies should be reserved for research settings and applied only in well-selected patients with clearly defined immune alterations. Until further evidence is available, the cornerstone of treatment remains the optimization of metabolic and endocrine factors, which may secondarily promote immune balance. In the future, immunomodulatory interventions may emerge as valuable adjuncts to improve implantation rates and live birth outcomes in women with PCOS.

## Conclusion

PCOS should be recognized not only as an endocrine–metabolic disorder but also as a systemic immunoinflammatory condition that directly affects reproductive performance. Immune dysregulation – including cytokine imbalance, altered lymphocyte subsets, and increased autoantibody production – contributes to implantation failure, miscarriage, and pregnancy complications. Although metabolic optimization remains the cornerstone of management, integrating immune evaluation into the assessment of women with PCOS may identify those at a greater reproductive risk. Lifestyle changes and the use of insulin sensitizers indirectly modulate immune dysfunction, whereas novel immunomodulatory strategies, although still investigational, hold promise for selected patients with defined immune phenotypes. A deeper understanding of the interaction between the metabolic, hormonal, and immune systems is essential for advancing precision medicine and improving fertility outcomes in this complex syndrome.

## Declaration of interest

The authors declare that there is no conflict of interest that could be perceived as prejudicing the impartiality of the work reported. Marcelo Borges Cavalcante is an Associate Editor of Reproduction and Fertility. Marcelo Borges Cavalcante was not involved in the peer-review process for this manuscript, on which he is listed as an author.

## Funding

This work was supported by Fundação Edson Queiroz – Edital 66/2024.

## Author contribution statement

TBR, DSS, and MBC contributed to the conceptualization and design of the review and participated in defining the methodology. TBR, DSS, LAAA, HAP, RERF, ALRM, MGRM, TGM, and LOM conducted the literature investigation and were responsible for writing the original draft of the manuscript. MBC supervised the project, performed the critical review and editing of the text, coordinated the research activities, and secured funding for the study. All authors contributed to the interpretation of findings, reviewed the final version of the manuscript, and approved it for submission.
